# Y and mitochondrial chromosomes in the heterogeneous stock rat population

**DOI:** 10.1093/g3journal/jkae213

**Published:** 2024-09-09

**Authors:** Faith Okamoto, Apurva S Chitre, Thiago Missfeldt Sanches, Denghui Chen, Daniel Munro, Allegra T Aron, Angela Beeson, Hannah V Bimschleger, Maya Eid, Angel G Garcia Martinez, Wenyan Han, Katie Holl, Tyler Jackson, Benjamin B Johnson, Christopher P King, Brittany N Kuhn, Alexander C Lamparelli, Alesa H Netzley, Khai-Minh H Nguyen, Beverly F Peng, Jordan A Tripi, Tengfei Wang, Kendra S Ziegler, Douglas J Adams, Amelie Baud, Lieselot L G Carrette, Hao Chen, Giordano de Guglielmo, Pieter Dorrestein, Olivier George, Keita Ishiwari, Monica M Jablonski, Thomas C Jhou, Marsida Kallupi, Rob Knight, Paul J Meyer, Leah C Solberg Woods, Oksana Polesskaya, Abraham A Palmer

**Affiliations:** Department of Psychiatry, University of California San Diego, La Jolla, CA 92093, USA; Bioinformatics and System Biology Program, University of California San Diego, La Jolla, CA 92093, USA; Department of Psychiatry, University of California San Diego, La Jolla, CA 92093, USA; Bioinformatics and System Biology Program, University of California San Diego, La Jolla, CA 92093, USA; Department of Psychiatry, University of California San Diego, La Jolla, CA 92093, USA; Skaggs School of Pharmacy and Pharmaceutical Sciences, University of California San Diego, La Jolla, CA 92093, USA; Department of Chemistry and Biochemistry, University of Denver, Denver, CO 80208, USA; Department of Internal Medicine, Molecular Medicine, Wake Forest University School of Medicine, Winston-Salem, NC 27157, USA; Department of Psychiatry, University of California San Diego, La Jolla, CA 92093, USA; Department of Neurology, Icahn School of Medicine at Mount Sinai, New York, NY 10029, USA; Department of Pharmacology, Addiction Science and Toxicology, University of Tennessee Health Science Center, Memphis, TN 38163, USA; Department of Pharmacology, Addiction Science and Toxicology, University of Tennessee Health Science Center, Memphis, TN 38163, USA; Department of Physiology, Medical College of Wisconsin, Milwaukee, WI 53226, USA; Department of Neurobiology, University of Maryland School of Medicine, Baltimore, MD 21201, USA; Department of Psychiatry, University of California San Diego, La Jolla, CA 92093, USA; Department of Psychology, University at Buffalo, Buffalo, NY 14260, USA; Department of Neuroscience, Medical University of South Carolina, Charleston, SC 29425, USA; Department of Psychology, University of California Los Angeles, Los Angeles, CA 90095, USA; Department of Emergency Medicine, University of Michigan, Ann Arbor, MI 48109, USA; Department of Psychiatry, University of California San Diego, La Jolla, CA 92093, USA; Department of Psychiatry, University of California San Diego, La Jolla, CA 92093, USA; Department of Psychology, University at Buffalo, Buffalo, NY 14260, USA; Department of Pharmacology, Addiction Science and Toxicology, University of Tennessee Health Science Center, Memphis, TN 38163, USA; Department of Psychiatry, University of California San Diego, La Jolla, CA 92093, USA; Department of Orthopedics, University of Colorado - Anschutz Medical Campus, Aurora, CO 80045, USA; Centre for Genomic Regulation, Barcelona Institute of Science and Technology, Barcelona, Spain; Universitat Pompeu Fabra, Barcelona, Spain; Department of Psychiatry, University of California San Diego, La Jolla, CA 92093, USA; Department of Pharmacology, Addiction Science and Toxicology, University of Tennessee Health Science Center, Memphis, TN 38163, USA; Department of Psychiatry, University of California San Diego, La Jolla, CA 92093, USA; Skaggs School of Pharmacy and Pharmaceutical Sciences, University of California San Diego, La Jolla, CA 92093, USA; Collaborative Mass Spectrometry Innovation Center, Skaggs School of Pharmacy and Pharmaceutical Sciences, University of California San Diego, La Jolla, CA 92093, USA; Department of Pharmacology, University of California San Diego, La Jolla, CA 92093, USA; Center for Microbiome Innovation, University of California San Diego, La Jolla, CA 92093, USA; Department of Psychiatry, University of California San Diego, La Jolla, CA 92093, USA; Department of Pharmacology and Toxicology, University at Buffalo, Buffalo, NY 14203, USA; Clinical and Research Institute on Addictions, University at Buffalo, Buffalo, NY 14203, USA; Department of Ophthalmology and Department of Anatomy and Neurobiology, University of Tennessee Health Science Center, Memphis, TN 38163, USA; Department of Neurobiology, University of Maryland School of Medicine, Baltimore, MD 21201, USA; Department of Psychiatry, University of California San Diego, La Jolla, CA 92093, USA; Department of Pediatrics, University of California San Diego, La Jolla, CA 92093, USA; Center for Microbiome Innovation, University of California San Diego, La Jolla, CA 92093, USA; Department of Computer Science and Engineering, University of California San Diego, La Jolla, CA 92093, USA; Department of Bioengineering, University of California San Diego, La Jolla, CA 92093, USA; Department of Psychology, University at Buffalo, Buffalo, NY 14260, USA; Department of Internal Medicine, Molecular Medicine, Wake Forest University School of Medicine, Winston-Salem, NC 27157, USA; Department of Psychiatry, University of California San Diego, La Jolla, CA 92093, USA; Department of Psychiatry, University of California San Diego, La Jolla, CA 92093, USA; Institute for Genomic Medicine, University of California San Diego, La Jolla, CA 92093, USA

**Keywords:** heterogeneous stock, rat, Y Chromosome, mitochondria, haplotype, low-coverage, PheWAS, RNA-seq

## Abstract

Genome-wide association studies typically evaluate the autosomes and sometimes the X Chromosome, but seldom consider the Y or mitochondrial (MT) Chromosomes. We genotyped the Y and MT Chromosomes in heterogeneous stock (HS) rats (*Rattus norvegicus*), an outbred population created from 8 inbred strains. We identified 8 distinct Y and 4 distinct MT Chromosomes among the 8 founders. However, only 2 types of each nonrecombinant chromosome were observed in our modern HS rat population (generations 81–97). Despite the relatively large sample size, there were virtually no significant associations for behavioral, physiological, metabolome, or microbiome traits after correcting for multiple comparisons. However, both Y and MT Chromosomes were strongly associated with the expression of a few genes located on those chromosomes, which provided a positive control. Our results suggest that within modern HS rats there are no Y and MT Chromosomes differences that strongly influence behavioral or physiological traits. These results do not address other ancestral Y and MT Chromosomes that do not appear in modern HS rats, nor do they address effects that may exist in other rat populations, or in other species.

## Introduction

Heterogeneous stock (HS) rats (*Rattus norvegicus*) are a well-established outbred population that have been used for genome-wide association studies (GWAS); yet, their Y and mitochondrial (MT) Chromosomes have been largely ignored. The Y Chromosome was poorly assembled in prior versions of the rat genome. This dramatically improved in a recent rat reference genome (mRatBN7.2). In contrast, the MT Chromosome was not updated in mRatBN7.2 (Tutaj *et al*. 2019; de Jong *et al*. 2024).

HS rats have been outbred for almost 100 generations. They were created in 1984 by intercrossing eight inbred strains: ACI/N, BN/SsN, BUF/N, F344/N, M520/N, MR/N, WKY/N, and WN/N (Hansen and Spuhler 1984). Modern HS rat genomes are mosaics of those 8 founder haplotypes (Solberg Woods and Mott 2017), which enables precise genetic mapping of complex traits (e.g. Johannesson *et al*. 2009; Baud *et al*. 2013; Chitre *et al*. 2020). However, as Y and MT are nonrecombinant, even in a modern HS rat, they are expected to be inherited in their entirety from a single founder; the Y Chromosome from the father and the MT from the mother.

Some Y and MT haplotyping methods cannot be used in HS rats. For example, we lack complete pedigrees that could have been used to trace expected Y or MT genotypes, as was done in collaborative cross (CC) mice (Broman 2022). We also lack curated lists of informative variants, as in human databases (e.g. Kloss-Brandstätter *et al*. 2011; Chen *et al*. 2021).

In humans, Y or MT haplogroups have been tested for association with many phenotypes (e.g. Jamain *et al*. 2002; Ma *et al*. 2014; Howe *et al*. 2017; Cai *et al*. 2021; Degenhardt *et al*. 2022). Replication has proved difficult; population structure confounds such work (Hagen *et al*. 2018). For example, schizophrenia was linked to MT in a Han Chinese (Wang *et al*. 2013) cohort, but not Spanish (Mosquera-Miguel *et al*. 2012) or Swedish (Gonçalves *et al*. 2018) cohorts.

Studies in CC mice found that Y or MT genotype was not associated with sex ratio (Haines *et al*. 2021), but was associated with the expression of genes located on the Y and MT Chromosomes (Keele *et al*. 2021). Mouse models designed for isolating genetic effects of Y (e.g. Martincová *et al*. 2019) and MT (e.g. Welch *et al*. 2023) found phenotypic associations, even suggesting transgenerational effects of paternal Y Chromosome genotype in daughters (Nelson *et al*. 2010). However, our review of the literature did not find comparable Y or MT analyses in outbred mice.

We identified variants that could be used to determine which founder had contributed the Y and MT to each individual HS rat. This approach is broadly similar to a prior study in DO mice (Chesler *et al*. 2016). We then tested for associations between Y and MT genotypes and a large collection of phenotypic data that have been collected over almost a decade of studies using HS rats (www.ratgenes.org). These data include behavioral, physiological, metabolome, microbiome and RNA-seq complex traits; in total, 12,055 rats were both haplotyped and phenotyped.

## Materials and methods

A Reagent Table is in the Supplementary Files.

### Genotype datasets

We used preexisting whole-genome sequencing (WGS) data from males representing each of the 8 founder strains (∼40× coverage). Single-nucleotide polymorphisms (SNPs) and indels on the Y and MT Chromosomes were called using GATK, as previously described (Chen 2022). We identified polymorphic sites in these data which distinguish the different founder Y and MT Chromosomes. We also used preexisting WGS data from 44 male and 44 female outbred HS rats (∼33× coverage); SNPs and indels were similarly called using GATK. Short tandem repeats (STRs) on the Y Chromosome were called in all WGS samples with HipSTR (Willems *et al*. 2017) and filtered with DumpSTR (Mousavi *et al*. 2020).

We also used preexisting low-coverage (∼0.25×) data from 15,120 outbred HS rats. These used double-digest genotyping-by-sequencing (ddGBS; Gileta *et al*. 2020) or low-coverage WGS (lcWGS; Chen *et al*. 2023) library preparation. Biallelic SNP genotypes on mRatBN7.2 were imputed by STITCH (Davies *et al*. 2016), as previously described (Chen *et al*. 2024). We did not use the variant filters previously described. Instead, we started with all variants produced by STITCH and then used custom filters to avoid excluding variants potentially useful to distinguish founder Y or MT (see “Genotype filters”). Because Y and MT are hemizygous, heterozygous calls are unexpected (Supplementary Fig. S1); when observed, those genotypes were treated as missing. All procedures prior to tissue collection were approved by the relevant Institutional Animals Care and Use Committees.

### Genotype filters

Our custom filters removed (1) variants with low INFO score (for low-coverage data), (2) variants with minor allele frequency (MAF) of 0, (3) variants with a high missing rate (>25%), and (4) individual samples with a high missing rate (>50%). We applied all or only some of these filters, always in this order, depending on the analysis. In particular, when visualizing by SNP to determine haplotypes (e.g. in alignments) we skipped the MAF filter to visualize fixed variants, and when plotting statistics (e.g. heterozygosity) by SNP in low-coverage data, we skipped all but the INFO score filter. Supplementary Fig. S2 shows the thresholds used against distributions of these statistics (INFO score, MAF = 0, per-SNP missing rate, per-sample missing rate) for low-coverage samples. We used these filters to create haplotype groups for association analyses.

### Unrooted trees

We applied all standard filters to high-coverage genotype data, then used a matrix of Hamming distance (scale of 0–1) pairwise ignoring missingness, i.e. removing variants missing in either sample. We created an unrooted neighbor-joining (NJ) tree (Talevich *et al*. 2012). These trees were used for understanding HS Y and MT phylogeny, but not for haplotype group-making.

### Statistical analysis

We performed a phenome-wide association study (PheWAS) for Y or MT haplotype via mixed linear model-based association (MLMA) analysis (Yang *et al*. 2014) with GCTA (Yang *et al*. 2011); see “GWAS phenotype association”. We tested normalized (cpm in edgeR) RNA-seq transcript abundance against Y or MT haplotype via a two-sample Wilcoxon rank-sums (i.e. Mann–Whitney) test (wilcox.test in R); see “Gene expression association”. We tested normalized RNA-seq transcript abundance against X Chromosome SNPs via a simple linear model (lm in R); see “*Dkc1* expression and X SNPs association”. We used the Benjamini & Hochberg (BH) false discovery rate (FDR) approach (p.adjust in R; Benjamini and Hochberg 1995). For a single, binary phenotype (one or two kidneys at birth), we tested for association with MT haplotype using a Fisher’s exact test (fisher.test in R).

### GWAS phenotype association

We used a genetic relationship matrix (GRM) constructed using PLINK (Chang *et al*. 2015) to account for autosomal (--chr 1-20) relatedness (Yang *et al*. 2010), which we expected to be correlated with Y and MT haplotype due to familial structure. After filtration by missingness (--geno 0.1), violations of Hardy–Weinberg equilibrium (--hwe 1e-10; Wigginton *et al*. 2005), and MAF (--maf 0.005), 5,315,011 SNPs and 15,120 samples remained. We fit a linear model on all raw values and covariates, then inverse-normal transformed the residuals.

The traits used for the PheWAS are shown in Supplementary Table S1, with their sample sizes and minor haplotype frequencies in Supplementary Fig. S3. We encoded Y and MT haplotypes as SNPs: reference-like haplotype (from the same haplogroup as BN) as homozygous reference allele (0), and other haplotype as homozygous alternate allele (2). We ran GCTA’s MLMA with these genotypes, the autosomal GRM, and processed phenotypes. We applied BH correction across all GWAS phenotypes, separately for Y and MT. We used FDR < 0.05 to define significance.

### Gene expression association

Our previous work mapping *cis* expression quantitative trait loci (eQTLs) showed a linear mixed model is unnecessary (Munro *et al*. 2022). Therefore, for computational simplicity, we approached gene expression analysis using methods standard in differential expression (DE) analysis, treating Y and MT haplotype as “conditions”, instead of eQTL mapping.

We used RNA-seq data processed using mRatBN7.2, presented as “log2” read count for all 10 tissues which were available from RatGTEx (Supplementary Table S2). The following filtering scheme was applied (separately for Y and MT): (1) samples with a haplotype assignment were retained, (2) for each tissue, genes that had detectable expression in less than 10% of samples were excluded.

We normalized counts using trimmed mean of M values (TMM; Robinson and Oshlack 2010), then used a Mann–Whitney test for DE. This test is robust to violation of a distribution (e.g. negative binomial) in large-sample DE analysis (Li *et al*. 2022). We again used FDR < 0.05<0.05 to define significance for all genes, in all tissues, for both the Y and MT Chromosomes.

A standard eQTL expression normalization method, which involves ranking genes within a sample (Munro *et al*. 2022), is nonoptimal for highly expressed genes, ranked highly in all samples. Ranking loses raw abundance information by introducing ties between ranks. Thus, we used TMM, which is a standard DE method (Corchete *et al*. 2020; Zhao *et al*. 2021).

### *Dkc1* expression and X SNPs association

For *Dkc1* (ENSRNOG00000055562), we tested for association between its TMM-normalized expression and SNPs on the X Chromosome. SNPs underwent the same filters as the GRM, except that only female rats were used for Hardy–Weinberg equilibrium tests. We used a simple linear model that regressed expression level against SNP genotype (0, 1, 2 copies of alternative allele), and, for male rats, Y haplogroup. We calculated *P*-values for all SNP associations where at least 5 rats had the minor allele. Associations were performed separately for each sex.

## Results

### Two major versions of Y are present in modern HS rats

**Fig. 1.**
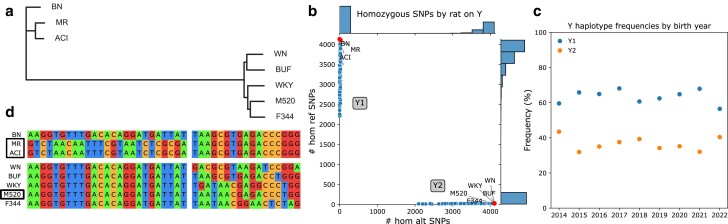
Y haplogroups present in HS founders and modern HS rats. a) NJ, unrooted tree using Y SNPs and indels in HS founders. Branch lengths correspond to genetic distance. b) Distribution of alleles by rat among Y SNPs passing filters (Supplementary Fig. S2). Plot shows count of reference alleles on *X*-axis and count of alternate alleles on *Y*-axis for each rat. Side plots are histograms of allele counts in modern HS rats (small dots). Missingness in low-coverage modern samples leads to scatter on the axes. Labeled large dots are HS founders. Y1 and Y2 haplogroups are labeled. c) Distribution of Y haplogroups in the HS rat population over time. Plot shows birth year on *X*-axis and haplogroup percentage on *Y*-axis. d) Pseudo-alignment of Y1 (top) or Y2 (bottom) founder haplotypes, at SNPs passing filters (Supplementary Fig. S2), where Y1 (left) or Y2 (right) founders have intragroup SNPs. Boxed founders have haplotypes present in modern HS rats.

All HS founders have distinct Y Chromosomes (Fig. 1a). BN, ACI, and MR are relatively similar to one another, and are also similar to the reference genome (which is based on BN), while the other five founders form a separate haplogroup.

We separated modern HS rats into two Y groups. We called 5,227 Y SNPs in 7,483 low-coverage samples from male modern HS rats. Coverage was generally low with a few spikes around the center (Supplementary Fig. S4). 4,132 SNPs and 7,471 samples remained after filtration by INFO score, MAF, and missingness (Supplementary Fig. S2a–d). SNPs in the pseudo-autosomal region are likely removed by these filters, as SNPs with high heterozygosity appear mid-filtration as having excess missingness. We grouped samples by whether they had more reference (Y1; 4,732 rats) or alternate (Y2; 2,739 rats) SNP alleles (Fig. 1b). Y1 is slightly more common in the modern male HS rat population (Fig. 1c).

Using STR data for a subset of 44 modern male HS rats that had sufficient coverage to call structural variants, we found ACI to be the most recent common ancestor of modern Y1 rats, while modern Y2 rats are closest to M520 (Supplementary Fig. S5).

We next found the consensus for each Y haplogroup. We use the same filters on the low-coverage samples, except for skipping MAF to retain newly fixed variants. We used these data to determine the consensus SNP genotypes for each haplogroup. The consensuses differ by 4,130 SNPs (S6A-C). We matched these consensuses to founders in their haplogroup; the results (boxed in Fig. 1d) agree with the founders identified using STR data.

There is negligible variation in SNP genotypes among Y1 rats (Supplementary Fig. S7a). Y2 has more variation; over 80 rats differ at one SNP (Supplementary Fig. S7b), possibly a mutation from the parent haplotype M520. We also identified a subgroup of Y1 with an apparent deletion of *Med14Y* (ENSRNOG00000060437) which low-coverage data cannot confidently call; thus, it was not used for association analyses (Supplementary File S1). Neither DNA-seq nor RNA-seq principal component analysis (PCA) revealed other groupings, except by library preparation method used for low-coverage sequencing (Supplementary Fig. S8a–b).

### Two versions of MT are present in modern HS rats

**Fig. 2.**
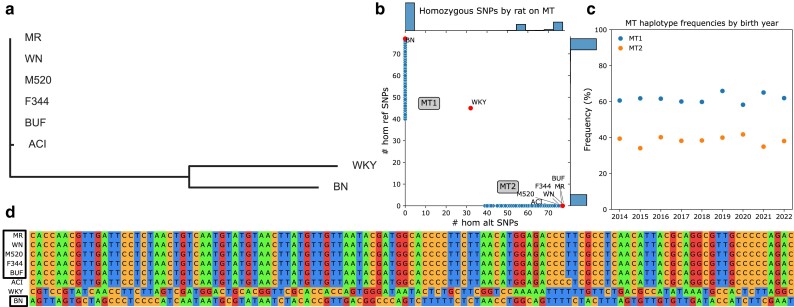
MT haplotypes present in HS founders and modern HS rats. a) NJ, unrooted tree using MT SNPs and indels in HS founders. Branch lengths correspond to genetic distance. b) Distribution of alleles by rat among MT SNPs passing filters (Supplementary Fig. S2). Plot shows count of reference alleles on *X*-axis and count of alternate alleles on *Y*-axis for each rat. Side plots are histograms of allele counts in modern HS rats (small dots). Missingness in low-coverage modern samples leads to scatter on the axes. Labeled large dots are HS founders. MT1 and MT2 haplogroups are labeled. c) Distribution of MT haplotypes in the HS rat population over time. Plot shows birth year on *X*-axis and haplotype percentage on *Y*-axis. d) Pseudo-alignment of founder haplotypes, at all MT SNPs called by low-coverage sequencing, colored by nucleotide. Boxed founders have haplotypes present in modern HS rats.

We found four MT haplotypes among the eight HS founders (Fig. 2a). BUF, F344, M520, MR, and WN share SNPs relative to BN, the basis of mRatBN7.2. WKY has a distinct MT haplotype, which was not observed among modern HS rats. The ACI haplotype is barely distinct from BUF, F344, M520, MR, and WN.

MT phylogeny of HS founders has been reported previously (Showmaker *et al*. 2020). However, their data (Ramdas *et al*. 2018) swapped WN and WKY. Our data puts WKY by itself, and WN in the large founder block with BUF, F344, M520, and MR. The Rat Genome Database (RGD; Vedi *et al*. 2023) variant visualizer (parameters: strains=HS founder strains, chromosome=MT, start=0, end=16,313) confirms the groups in Fig. 2. Complete MT genome sequencing of inbred substrains related to four of the HS founders (ACI/Eur, BN/NHsdMcwi, F344/NHsd, and WKY/NCrl) found the same relationships (Schlick *et al*. 2006).

We separated modern HS rats into MT groups. We called 117 MT SNPs in 15,120 low-coverage samples from modern HS rats, with higher coverage in lcWGS samples and several defined peaks of coverage in ddGBS samples (Supplementary Figs. S9, S10). 77 SNPs and 14,971 samples remained after filtration by INFO score, MAF, and missingness (Supplementary Fig. S2e-h). We grouped samples by whether they had more reference (MT1, 9,287 rats) or alternate (MT2, 5,684 rats) SNP alleles (Fig. 2b). MT1 is somewhat more common in the modern HS rat population (Fig. 2c).

We confirmed these as the only two MT haplotypes present in the modern low-coverage SNP genotypes. All modern HS rat MT match at least one of two SNP haplotypes, ignoring missingness. These two modern MT haplotypes are separated by 77 SNPs (Supplementary Fig. S6d–f). Each modern MT matches an ostensibly extant founder haplotype (Fig. 2d). Neither DNA-seq or RNA-seq PCA revealed further groupings, except by library preparation method (Supplementary Fig. S8c-d).

### Y haplogroup is associated with Y gene expression

**Fig. 3.**
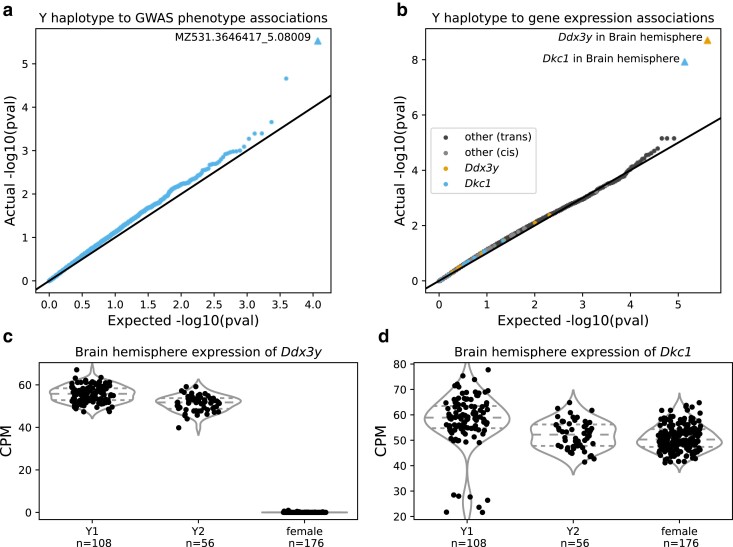
Results of Y haplogroup association tests. a–b) QQ plots of a) MLMAs between Y haplogroup and GWAS phenotypes and b) Mann–Whitney tests between Y haplogroup and gene expression. Each dot represents a single trait (in b, a single gene in a single tissue, colored by gene). Plot shows actual distribution of unadjusted *P*-values on *Y*-axis, against expected distribution (null hypothesis of no association) on *X*-axis. Significant associations (FDR < 0.05) are triangles. c–d) Effect plots of c) *Ddx3y* and d) *Dkc1* expression in the brain, split by Y haplogroup, with females for context. Horizontal lines show quantiles. Plots show each sample’s normalized CPM on *Y*-axis; samples are split into Y haplogroups on *X*-axis. Q-values in Supplementary Table S3.

We investigated the effect of Y haplogroup on various phenotypes. Y haplogroup was not significantly associated with any of the phenotypes examined (Fig. 3a), except for levels of MZ531.3646417_5.08009 (Supplementary Fig. S11), an unannotated metabolite that was measured in the cecum. Y haplogroup was associated with expression of *Ddx3y* and *Dkc1*, both of which are located on the Y Chromosome (Fig. 3b–d, Supplementary Table S3).

*Ddx3y* is an RNA helicase. In humans, it is involved with neuron development in males (Vakilian *et al*. 2015). Its male-specificity is sometimes used for determining sex, e.g. in humans (Hoch *et al*. 2020) and pigs (Teixeira *et al*. 2019). Consistent with this application, we found that *Ddx3y* was not expressed in female rats (Fig. 3c).

Mutations in *Dkc1*’s human ortholog cause X-linked dyskeratosis congenita (Heiss *et al*. 1998); many orthologs of this gene are on the X Chromosome (RGD). In mRatBN7.2 *Dkc1* is on an unplaced Y Chromosome contig; in the most recent rat reference genome, GRCr8, it is on the X Chromosome (RGD). Unlike *Ddx3y*, *Dkc1* is expressed in females (Fig. 3d). This suggests *Dkc1* is in the pseudo-autosomal region. However, the rat pseudo-autosomal region, which has lost many genes to autosomes (Maxeiner *et al*. 2020), does not have well-established boundaries (Raudsepp and Chowdhary 2016). We did not find strong evidence of linkage between SNPs on the X Chromosome and *Dkc1* expression (Supplementary Fig. S12), which would have supported its localization to the pseudo-autosomal region. The subgroup of Y1 rats with low *Dkc1* expression is more readily explained by a related presence–absence variant (File S1).

### MT haplotype is associated with gene expression

**Fig. 4.**
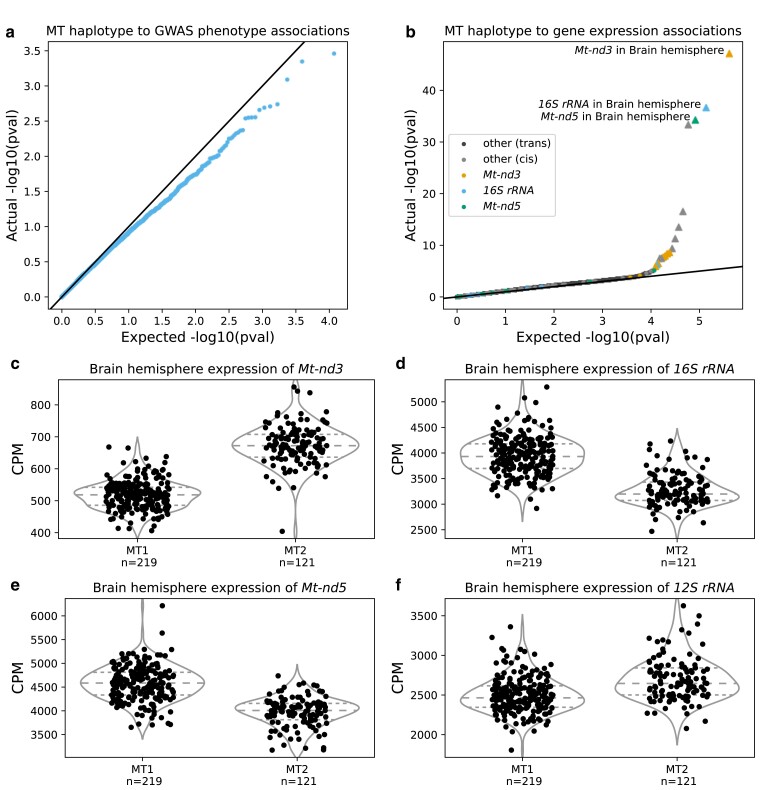
Results of MT haplotype association tests. a–b) QQ plots of a) MLMAs between MT haplotype and GWAS phenotypes and b) Mann–Whitney tests between MT haplotype and gene expression. Each dot represents a single trait (in b, a single gene in a single tissue, colored by gene). Plot shows actual distribution of unadjusted *P*-values on *Y*-axis, against expected distribution (null hypothesis of no association) on *X*-axis. Significant associations (FDR < 0.05) are triangles. c–f) Representative effect plots for significant associations, of c) *Mt-nd3*, d) *16S rRNA*, e) *Mt-nd5*, and f) *12S rRNA* expression in the brain, split by MT haplotype. Plots show each sample’s normalized CPM on *Y*-axis; samples are split into MT haplotypes on *X*-axis. Effect plots for all significant associations with MT haplotype are shown in Supplementary Fig. S13. *Q*-values in Supplementary Table S4.

We investigated the effect of MT haplotype on all available phenotypes; none of the results were significant (Fig. 4a), despite sufficient sample size and minor haplotype frequency for good power (Supplementary Fig. S3). In addition, we separately tested for association with having one or two kidneys at birth, as Showmaker *et al*. (2020) hypothesized an effect from BN (MT1) vs. ACI (similar to MT2) MT genotype. MT1 rats have a higher rate of being born with a single kidney (Supplementary Table S5) but a one-sided Fisher’s exact test against MT haplotype was insignificant (P=0.14). However, MT haplotype was associated with the expression of several MT genes (Fig. 4b–f, Supplementary Fig. S11, Table S4).

Complex I is the first enzyme in the electron transport chain. In 7 of 10 tissues tested, its *Mt-nd3* subunit is up-regulated in MT2 relative to MT1. Every other MT-encoded subunit (*Mt-nd1*, *Mt-nd2*, *Mt-nd4*, *Mt-nd4l*, *Mt-nd5*, *Mt-nd6*) is down-regulated in MT2, resulting in different subunit ratios. Also, both MT-encoded ribosomal RNAs have significant DE, which are possible artifacts of imperfect poly-A tail selection.

## Discussion

We performed a large-scale study to identify phenotypes influenced by the nonrecombinant Y and MT Chromosomes in 12,055 HS rats. The 8 founders of the HS population had two major Y Chromosome (Fig. 1a) and three major MT Chromosome (Fig. 2a) haplotype groups. In modern HS rats, we observed two Y haplogroups (Y1 and Y2; Fig. 1b), with minimal intragroup variation. Similarly, in modern HS rats we observed two MT haplotypes (MT1 and MT2; Fig. 2b), with no genotyped intragroup variation.

We assigned 12,055 rats who were both phenotyped (n phenotyped=12,116) and haplotyped (n halotyped=15,042) to Y1 or Y2 (for males) and MT1 or MT2 haplotypes and then sought to identify associations with an array of behavioral and physiological phenotypes. Remarkably, there were virtually no significant associations (Figs. 3a, 4a, Supplementary Table S5). For both Y and MT, we identified *cis*-located genes with DE between haplogroups (Figs. 3b, 4b). While these eQTLs do not cause detectable changes in the behavioral and physiological traits we studied, they provide an important positive control, demonstrating that we can accurately call Y and MT haplotypes. Overall, our results show that previous genetics studies in HS rats which did not examine the Y and MT Chromosomes, did not overlook important genetic effects.

A strength of our study is the fact that the genetic structure of HS rats makes them well suited for studying Y and MT. Whereas human studies can be confounded by correlations between MT and nuclear genotype (Hagen *et al*. 2018), the HS breeding strategy (Solberg Woods and Mott 2017) and our use of MLMA for PheWAS avoided these problems. In addition, all of the observed Y or MT haplotypes are very common (Figs. 1c, 2c), unlike the situation in DO mice (Chesler *et al*. 2016) or humans (Howe *et al*. 2017), providing good power to detect associations.

Our results indicate that only a few of the founder Y and MT Chromosomes have persisted into modern HS rats. There are possibly further subtle Y and MT variations which our data could not distinguish. The lost haplotypes could reflect genetic drift or inadvertent selection due to differences in fitness or fecundity; our data can not distinguish between these two possibilities. Thus, it is possible that some of the unobserved Y and MT Chromosomes would have shown phenotypic consequences had they been present among the modern HS rats that we studied. For example, rats with comparable nuclear genotypes and a BN or WKY MT differ on various physiological traits (Sathiaseelan *et al*. 2023). However, as WKY’s MT is not observed in modern HS rats, we were unable to test for its effect on physiological traits.

In summary, we describe Y and MT haplotype structure in modern HS rats, and present results from well-powered association analyses with various phenotypes. Haplotypes are inherited from specific HS founders and cause differential expression of several genes of biological importance, including Complex I subunits and genes with orthologs to human sex-linked disorders. Methods described here may be extended to other rat populations for further investigation of Y and MT.

## Supplementary Material

jkae213_Supplementary_Data

## Data Availability

HS rats are available at https://ratgenes.org/cores/core-b/. All data required to reproduce these analyses, and raw results (with unadjusted *P*-values) from association tests, are in UC San Diego Library Digital Collections with DOI 10.6075/J0VX0GQQ. (Filename key in File S2.) Code to reproduce these analyses is in Zenodo with DOI 10.5281/zenodo.11493119, and in GitHub. Low-coverage raw reads are in the NCBI SRA with accession PRJNA1022514. Low-coverage autosomal genotypes used for the GRM are in UCSD Library with DOI 10.6075/J00G3KBX. Low-coverage genotyping was conducted at the IGM Genomics Center. Supplemental material available at G3 online.
