# Supplementary material for: Y and mitochondrial chromosomes in the heterogeneous stock rat population
Source: G3 (Bethesda). 2024 Sep 9;14(11):jkae213. doi: 10.1093/g3journal/jkae213 (PMC11540319; doi:10.1093/g3journal/jkae213)
Supplement: jkae213_Supplementary_Data [file jkae213_supplementary_data.zip › File_S1_G3-2024-404832.pdf]

Deletion of *Med14Y* in the HS rat population

This document refers to *Med14Y*. Alternate gene identifiers include ENSRNOG00000060437, LOC103694537, and mediator of RNA polymerase II transcription subunit 14-like.

Introduction

Med14Y is a 28kbp gene on the *R. norvegicus* Y Chromosome. In mRatBN7.2 it is on an unplaced Y Chromosome contig (NW_023637718.1). This contig includes *Sry* copies, and, at 93kbp distal, *Dkc1* (ENSRNOG00000055562). In GRCr8 *Med14Y* is on the main Y contig (RefSeq; O'Leary *et al*. 2016). *Med14Y* resulted from a retrotransposition (Prokop *et al*. 2013).

Med14Y appears to be within a presence/absence variant (PAV) in male heterogeneous stock (HS) rats. This variant has three consequences which are seen in available data.

1. DNA-seq depth along *Med14Y* is low/0.
2. RNA-seq expression of *Med14Y* is low/0.
3. RNA-seq expression of *Dkc1* is low compared to normal males.

All reads (DNA-seq and RNA-seq) were aligned to mRatBN7.2, and thus an unplaced contig. As variants were not called on unplaced contigs, the above points are used to call this PAV.

RNA evidence for *Med14Y* deletion

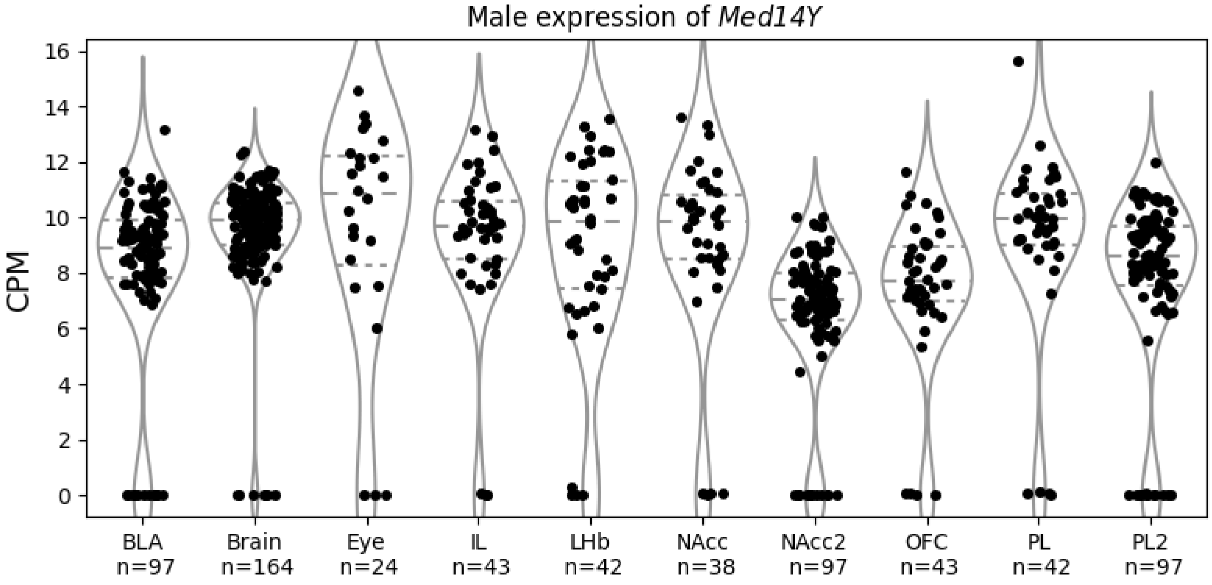

Figure A. Expression of *Med14Y*. Males from all available RNA-seq datasets used. Short names of tissue datasets (see Table S2) on X-axis, expression on Y-axis. Quantile lines shown.

Modern HS rats (generations 73-80) underwent RNA-seq (see RatGTEx or “Gene expression association” from main text). Most males have significant *Med14Y* expression, but some have essentially 0 (Figure A). There are two rats, 000789990A and 000792A1F9, whose Eye samples have low expression of *Med14Y*, though their Brain samples have normal expression. As these rats are clearly capable of expressing *Med14Y*, their lack of Eye expression cannot be due to a deletion of the gene, but may be instead due to some other rare effect which modulates expression in a tissue-specific manner. Another possibility is that samples were mixed up or contaminated, however after reviewing the data, we do not think this is the case.

Modern HS rats have two Y haplotypes groups (see “Two major versions of Y are present in modern HS rats” from main text). All 24 rats with consistently low *Med14Y* expression have type Y1. These data suggest a mutation in a Y1 HS rat generations ago.

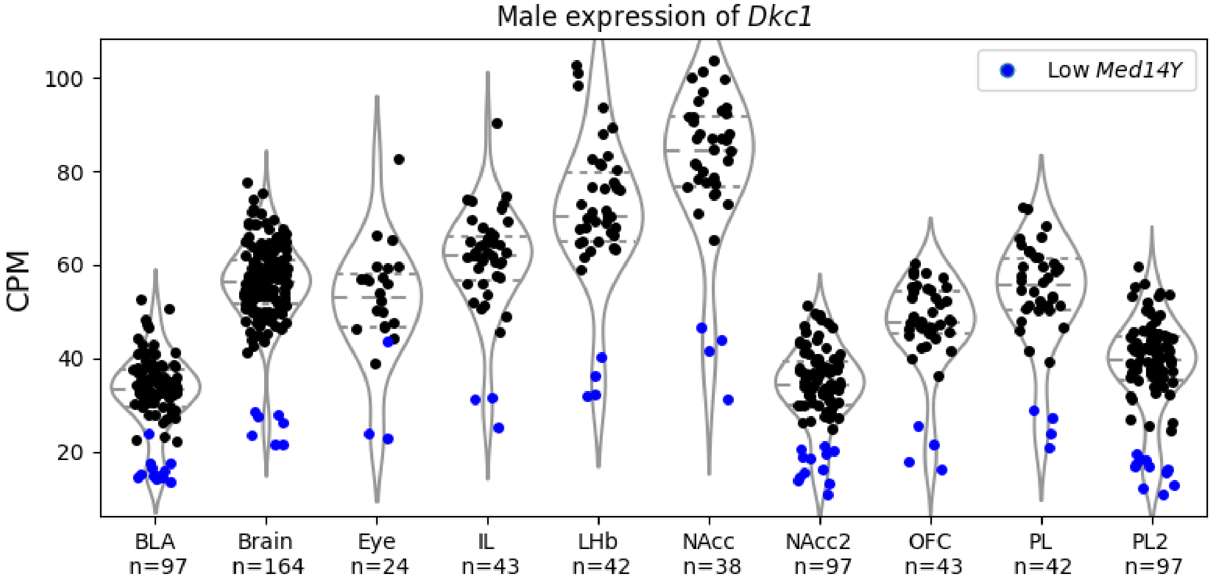

Figure B. Expression of *Dkc1* by PAV of *Med14Y*. Males from all available RNA-seq datasets used. Short names of tissue datasets (see Table S2) on X-axis, expression on Y-axis. Quantile lines shown. Samples with low expression of *Med14Y* colored blue.

Dkc1, a gene located on the same contig as *Med14Y* in mRatBN7.2, has markedly decreased expression in Y1 rats with *Med14Y* deletion (Figure B). (The rat with relatively normal *Dkc1* expression in Eye despite low *Med14Y* is 000789990A, one of the rats with inconsistent expression.) Notably, this explains the subgroup seen in main-text Figure 3D: most Y1 rats have higher brain hemisphere expression of *Dkc1* than Y2 rats, except for a Y1 subgroup with low expression. The mechanism of *Dkc1* downregulation is unclear but may involve deletion of regulatory sequences or loss of direct action by the *Med14Y* protein.

DNA evidence for *Med14Y* deletion

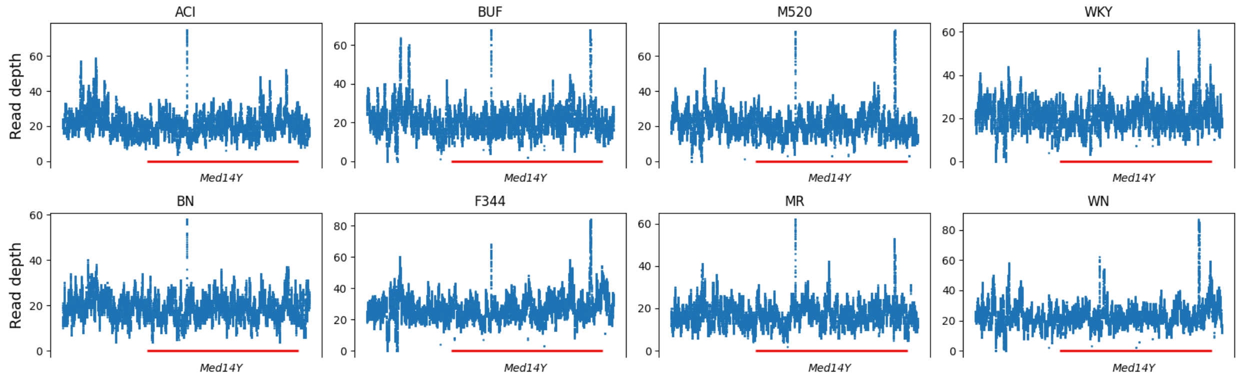

Figure C. Read depth in founder strains around *Med14Y*. Position on X-axis, depth on Y-axis.

HS rats are descended from eight inbred strains (Hansen and Spuhler 1984). Deeply-sequenced WGS samples from male rats demonstrate significant read depth within *Med14Y* in all founder strains (Figure C). Thus, a lack of *Med14Y* in modern HS rats is due to a *de novo* deletion.

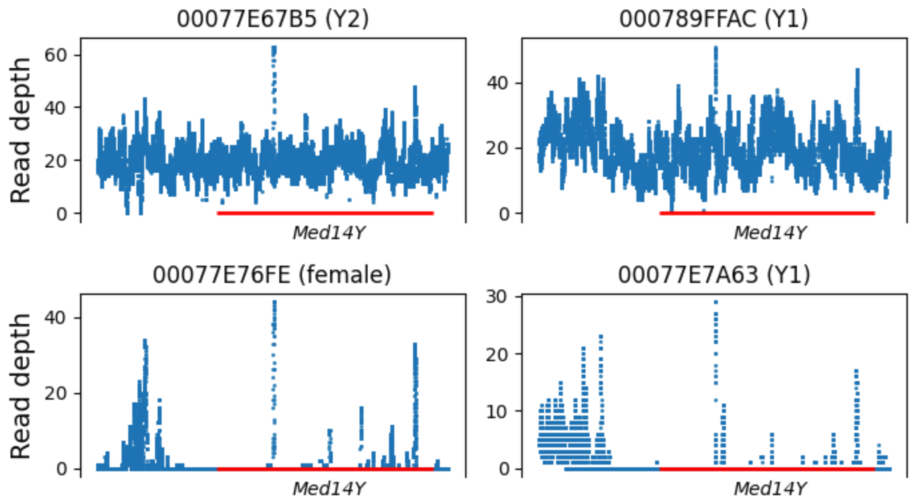

Figure D. Read depth in modern HS rats around *Med14Y*. Position on X-axis, depth on Y-axis.

Five of the RNA-seq datasets (IL, LHb, NAcc, OFC, PL) included the modern HS rats with deep WGS DNA-seq data (see "Genotype datasets" from main text). These tissue datasets have a subgroup with low *Med14Y* expression (Figure A), which always is 00078A01A6, 00077EA7E6, 00078A0058, and 00078A2463, except when one was removed during RNA-seq quality control. 00078A01A6 had poor-quality WGS data and was thus removed from the DNA-seq data. The other rats with RNA evidence for *Med14Y* PAV, and 00077E7A63 (which lacks RNA-seq data), have corresponding DNA evidence; their DNA-seq read depth in this region is comparable to a female (expected to lack *Med14Y* due to lacking a Y Chromosome), instead of the steady coverage seen in males. Coverage in samples from representative rats is shown in Figure D.

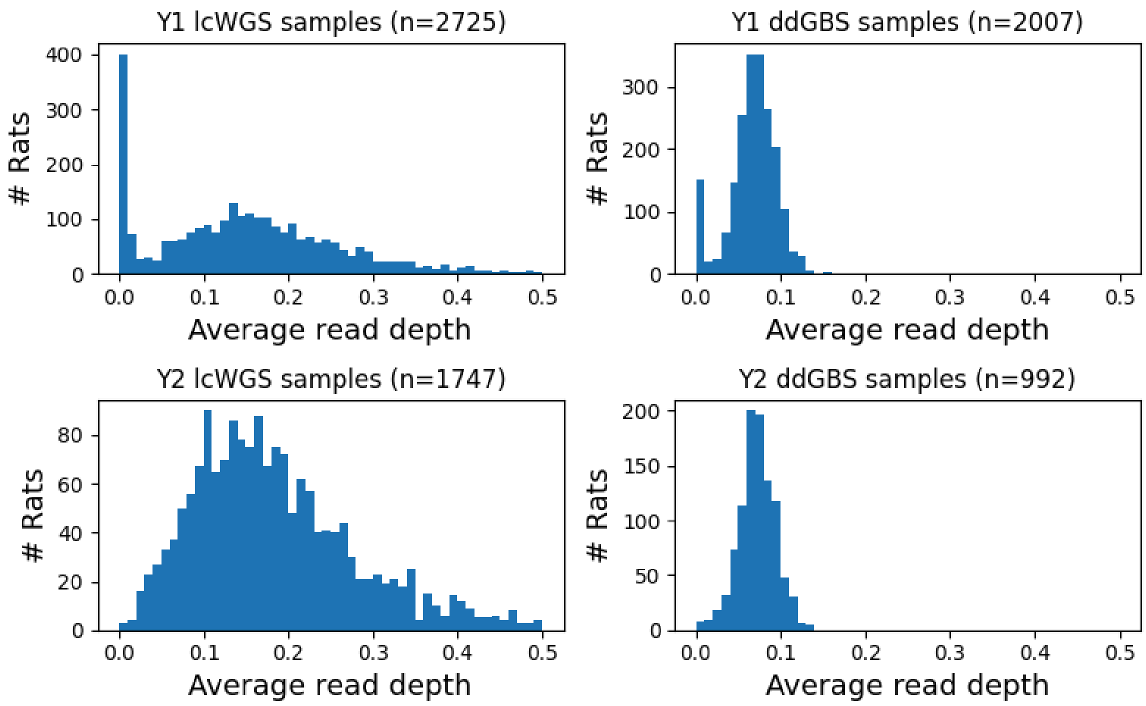

Figure E. Histograms of average read depth in samples from modern HS rats within *Med14Y*'s annotation. Average depth on X-axis, number of rats on Y-axis. Outliers with average depth of over 0.5 cut off. Bins of size 0.01. Samples split by Y haplotype and library preparation method.

In low coverage DNA-seq data from modern HS rats (see "Genotype datasets" from main text), average read depth along *Med14Y* is somewhat normally distributed, except for a peak around 0 within Y1 rats (Figure E). A significant subgroup of Y1 rats (due to an ancestral mutation) with a deletion of *Med14Y* explains the lack of coverage among a subset of Y1 rats. This subgroup is more prevalent in lcWGS rats, the sequencing method used for more recent generations. However, due to general low coverage, the evidence for any specific rat's PAV variant is weaker.

Conclusion

A *de novo* PAV, involving deletion of *Med14Y* and lower *Dkc1* expression, occurred an unknown number of generations ago in Y1 rats; this PAV is not observed in any of the founders. This PAV is somewhat common in the Y1 haplotype within modern male HS rats.

References

Hansen C, Spuhler K. 1984. Development of the National Institutes of Health Genetically Heterogeneous Rat Stock. Alcohol: Clinical and Experimental Research. 8(5):477–479. doi:10.1111/j.1530-0277.1984.tb05706.x.

O'Leary NA, Wright MW, Brister JR, Ciufo S, Haddad D, McVeigh R, Rajput B, Robbertse B, Smith-White B, Ako-Adjei D, et al. 2016. Reference sequence (RefSeq) database at NCBI: current status, taxonomic expansion, and functional annotation. Nucleic Acids Res. 44(D1):D733-745. doi:10.1093/nar/gkv1189.

Prokop JW, Underwood AC, Turner ME, Miller N, Pietrzak D, Scott S, Smith C, Milsted A. 2013. Analysis of Sry duplications on the Rattus norvegicus Y-chromosome. BMC Genomics. 14(1):792. doi:10.1186/1471-2164-14-792.
