# Supplementary material for: Y and mitochondrial chromosomes in the heterogeneous stock rat population
Source: G3 (Bethesda). 2024 Sep 9;14(11):jkae213. doi: 10.1093/g3journal/jkae213 (PMC11540319; doi:10.1093/g3journal/jkae213)
Supplement: jkae213_Supplementary_Data [file jkae213_supplementary_data.zip › Supplemental_Figures_and_Tables_G3-2024-404832.pdf]

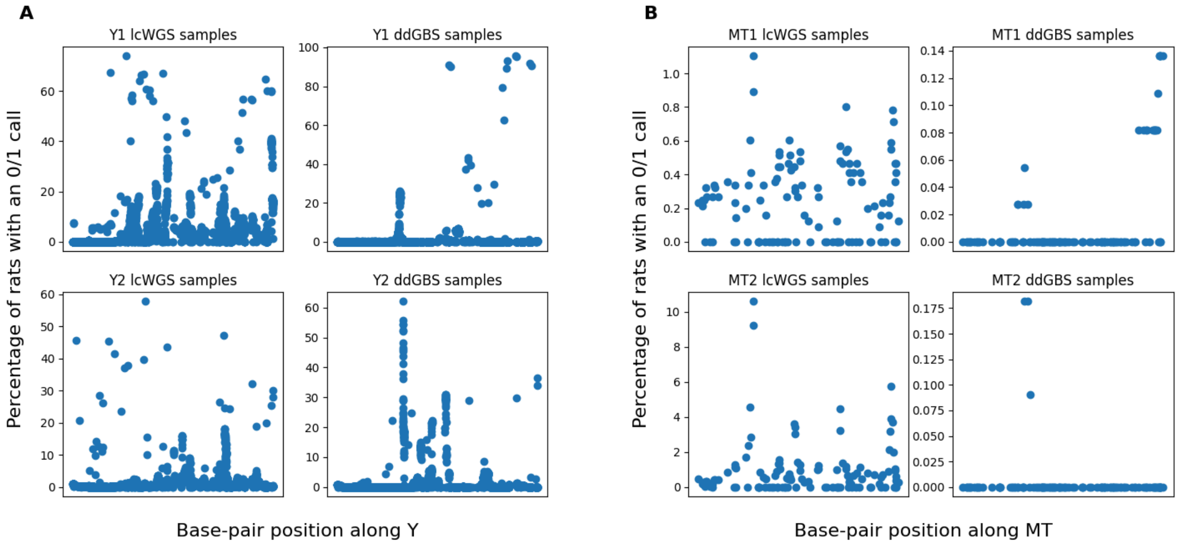

Figure S1. Heterozygous calls in low-coverage Y and MT chromosomes are meaningless. **A-B.** Frequency of heterozygosity, split by haplogroup and library preparation method. Plot shows SNP position along the chromosome on X-axis and percentage of rats with an 0/1 genotype call on Y-axis. Note that SNPs with heterozygosity are more dependent on sequencing method than haplogroup; if heterozygosity distinguished a true subgroup, we would expect a focused set of heterozygous calls across both sequencing methods for a single haplotype.

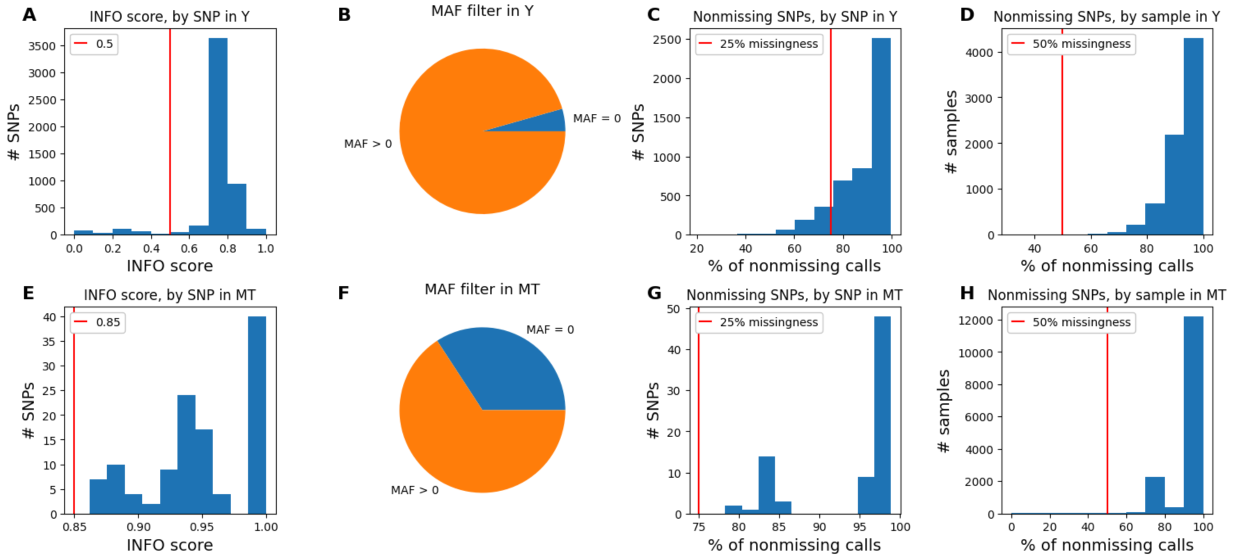

Figure S2. Filtration of raw low-coverage imputation output. Filters were applied from left to right; e.g. SNPs filtered out for low INFO score were not included in MAF filtration, and SNPs filtered out for by-SNP missingness were not included in calculations of by-rat missingness. Vertical lines correspond to thresholds used. **A.** A filter of INFO score ≥ 0.85 (threshold chosen to allow all MT SNPs) removed no MT SNPs. **B.** A filter of MAF > 0 removed 40 MT SNPs **C.** A filter of by-SNP missingness $\leq 25\%$ removed no MT SNPs. **D.** A filter of by-rat missingness $\leq$ 50% removed 149 rats. **E.** A filter of INFO score ≥ 0.5 (threshold chosen to be past the peak of good SNPs) removed 300 Y SNPs. **F.** A filter of MAF > 0 removed 217 Y SNPs **G.** A filter of by-SNP missingness $\leq 25\%$ removed 578 Y SNPs. **H.** A filter of by-rat missingness $\leq 50\%$ removed 12 rats.

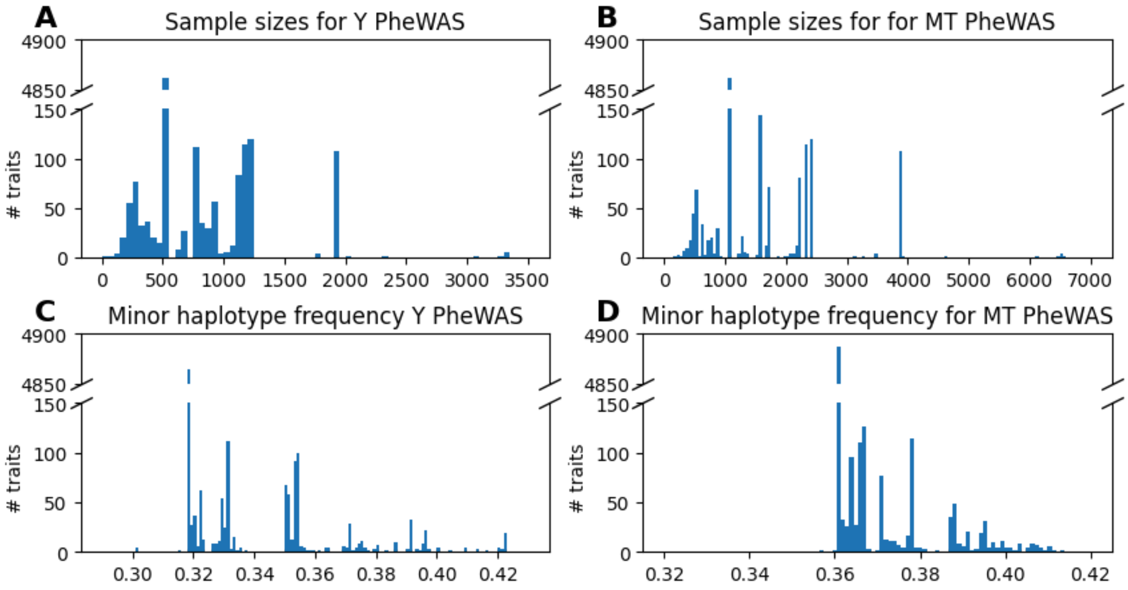

Figure S3. Characterization of samples used in GWAS phenotype tests. **A-B.** Histograms of sample size for each test against the **A.** Y and **B.** MT Chromosomes. Binwidth of 50. Rats dropped from GWAS phenotype tests for lacking a haplogroup; notably, all female rats lack a Y haplogroup. **C-D.** Minor haplotype frequency (similar to minor allele frequency) for each test against the **C.** Y and **D.** MT Chromosomes. Binwidth of 0.005. The peak noticeable in all panels is due to the project "Genetic Basis of Cecum Metabolome Composition in Heterogeneous Stock Rats", which contributes over 4,800 of the 5,850 phenotypes tested (Table S1).

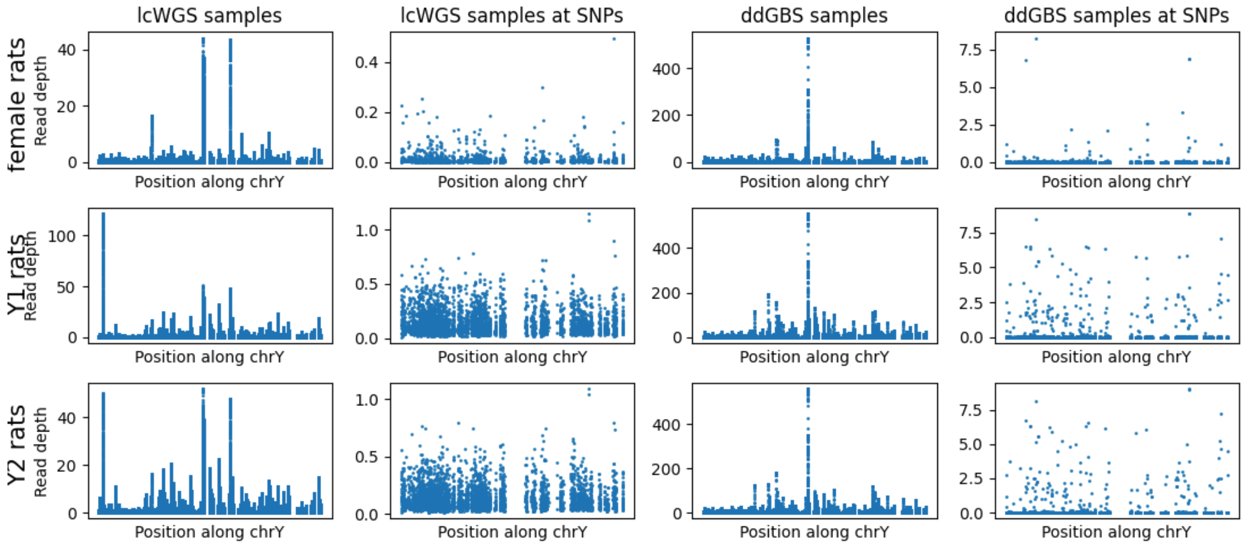

Figure S4. Average read depth in low-coverage data along the Y Chromosome. Plot shows position along Y on X-axis and average read depth on Y-axis. Samples split by Y haplogroup and by library preparation method. Positions with SNPs between Y1 and Y2 (Figure S6) plotted separately.

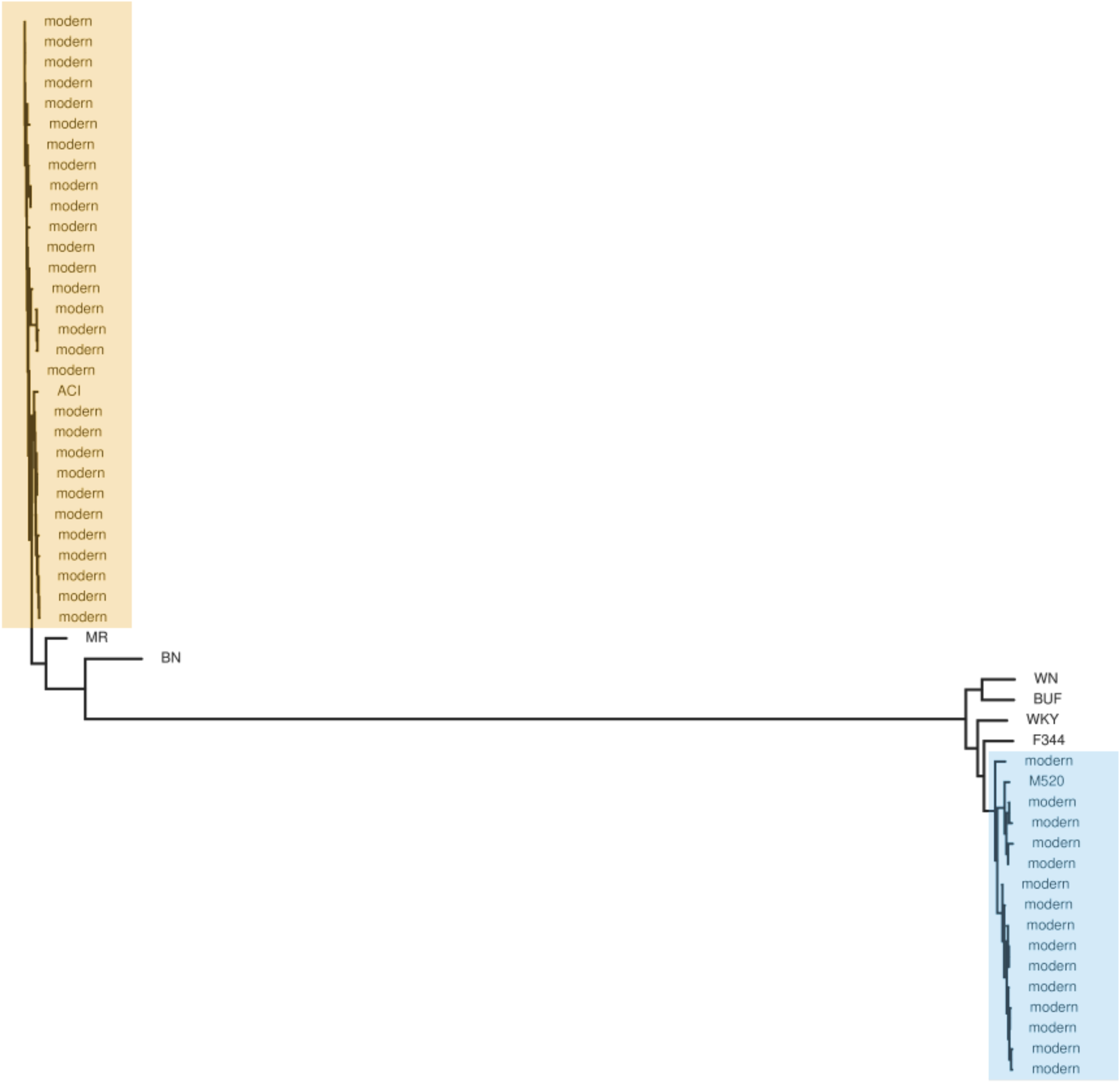

Figure S5. Similarity between Y STR genotypes. NJ, unrooted tree using Y STRs in founders and 44 (29 Y1, 15 Y2) deeply sequenced modern male HS rats. Branch lengths correspond to genetic distance. Modern clades highlighted, each including a single ostensible donor founder.

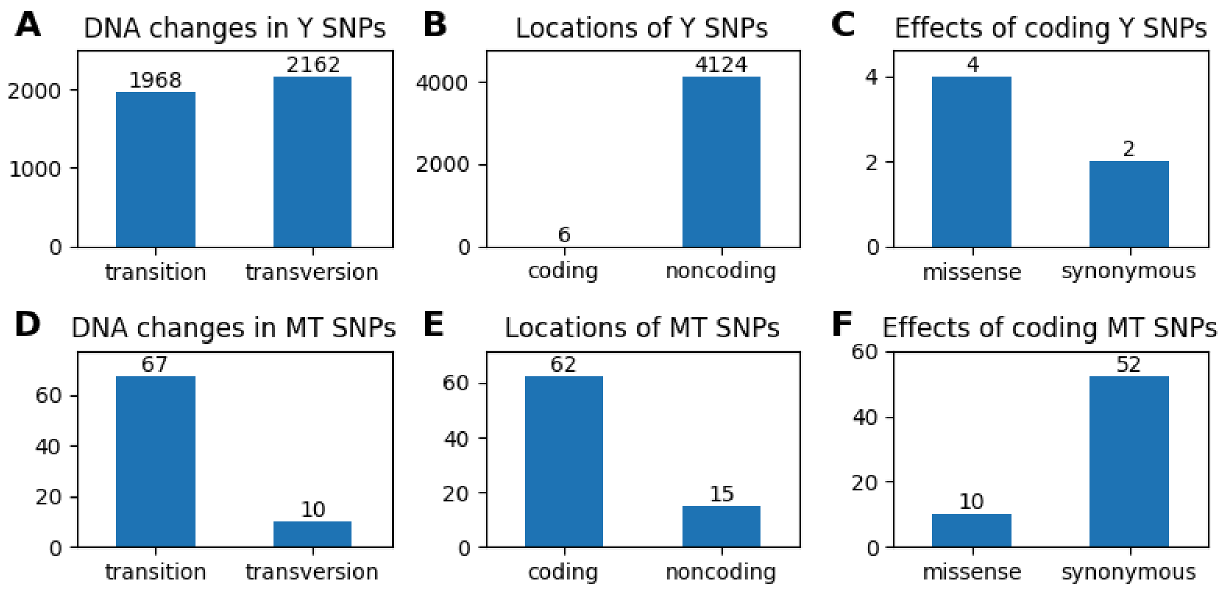

Figure S6. Characterization of SNPs separating haplotypes. Bar charts for SNPs between **A-C.** Y1 and Y2 or **D-F.** MT1 and MT2 with locations and effect relative to gene annotations from mRatBN7.2. Bars labeled by height.

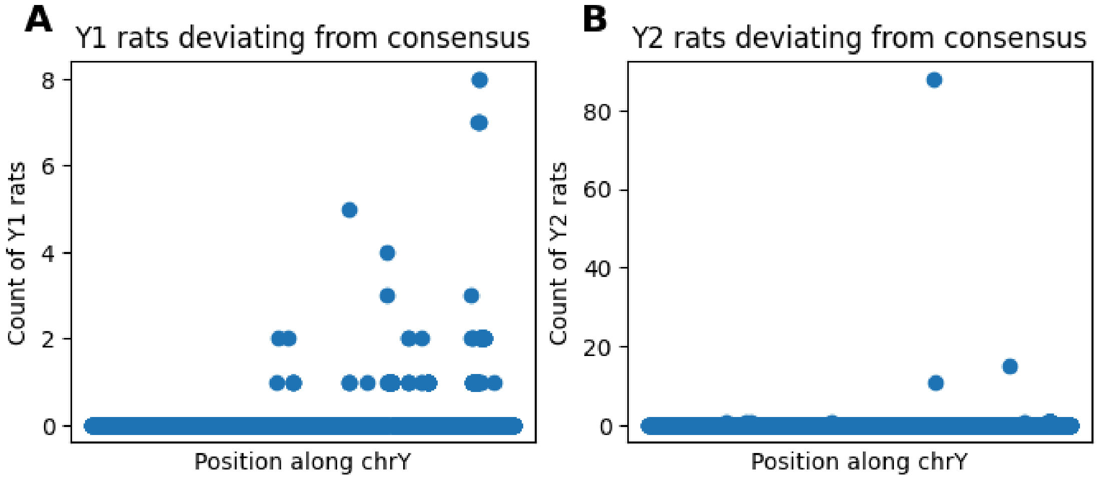

Figure S7. Deviation from haplogroup consensus SNP genotypes. **A-B.** Number of modern **A.** Y1 or **B.** Y2 low-coverage SNP genotypes deviating from their modern haplogroup's consensus. Plot shows SNP position along the Y Chromosome on X-axis and number of low-coverage genotypes different from the haplotype on Y-axis.

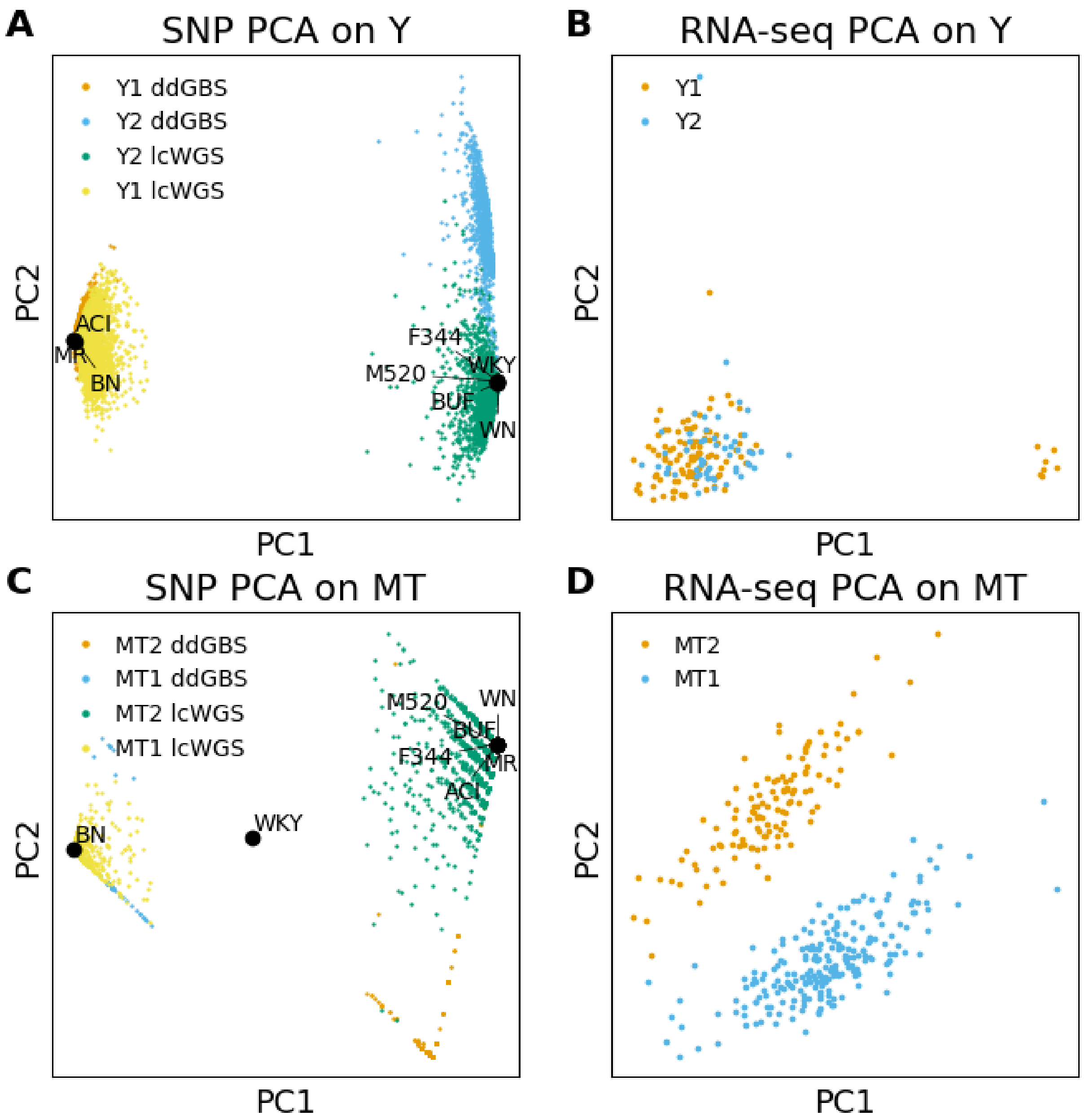

Figure S8. PCA does not reveal further genetic groupings, but does show an effect of library preparation method. **A.** Biplot of PCA using Y SNPs filtered as was done to make haplogroups (Figure S2). SNPs encoded as 0 (reference) or 1 (alternate), with mean imputation for missing values. Plot shows PC1 on X-axis and PC2 on Y-axis. Modern rats colored by haplogroup and library preparation method. HS founders are large, labeled black dots, projected on the same PCs. **B.** Biplot of PCA using RNA-seq transcript abundance in brain hemisphere data ("Brain" in Table S2) of genes on Y with expression in >50% of samples. Expression levels normalized via dividing by maximum expression for each gene. Plot shows PC1 on X-axis and PC2 on Y-axis. Modern rats colored by haplogroup. Side group of Y1 rats have a PAV affecting *Dkc1* and *Med14Y* expression; see File S1 **C.** Biplot of PCA using MT SNPs done the same as for Y. **D.** Biplot of PCA using MT genes done the same as for Y.

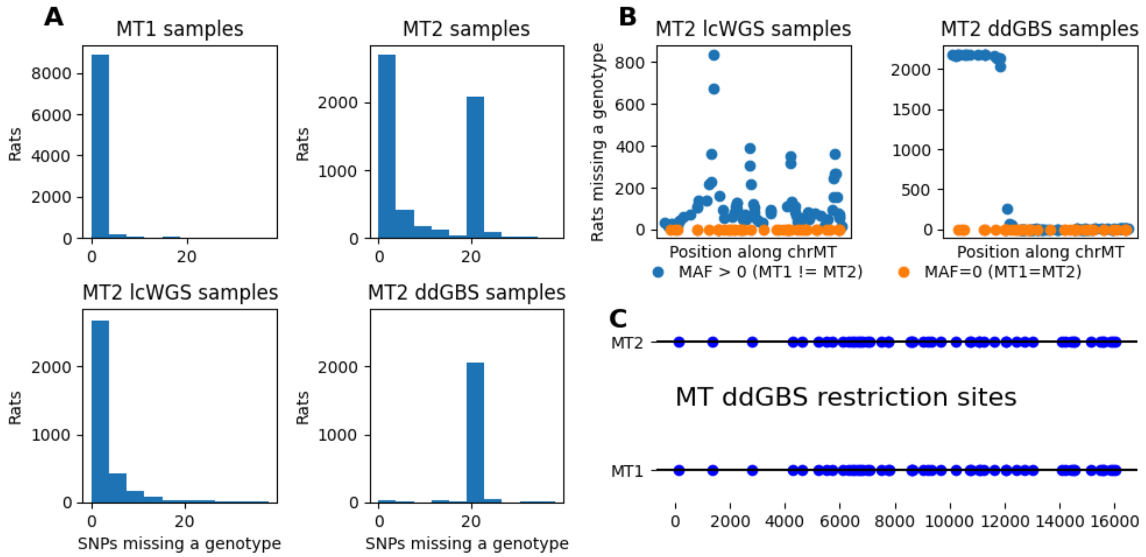

Figure S9. MT missingness patterns. Distribution of MT2 missingness is bimodal, due to a group of SNPs missing in ddGBS samples. This block of SNPs is differentially imputed: both MT haplotypes lack the necessary distribution of restriction sites, and thus have essentially no coverage. However, the reference MT1, and all SNPs with MAF=0 between MT1 and MT2 (i.e. all reference alleles), were imputed. **A.** Histograms of per-rat missingness for samples with MT1 and MT2 haplotypes, and for MT2 samples split by library preparation method used. **B.** Missingness across the MT chromosome for samples with the MT2 haplotype, shown for the lcWGS and ddGBS library preparation methods. Plot shows SNP position along the MT chromosome on X-axis and count of MT2 rats missing a genotype on Y-axis. Orange dots are SNPs identical between MT1 and MT2, while blue dots show SNPs which vary between haplotypes. **C.** Locations of ddGBS restriction sites. AY172581.1 is used for MT1. MT2 is mutated based on MT SNP and indel variants in founders.

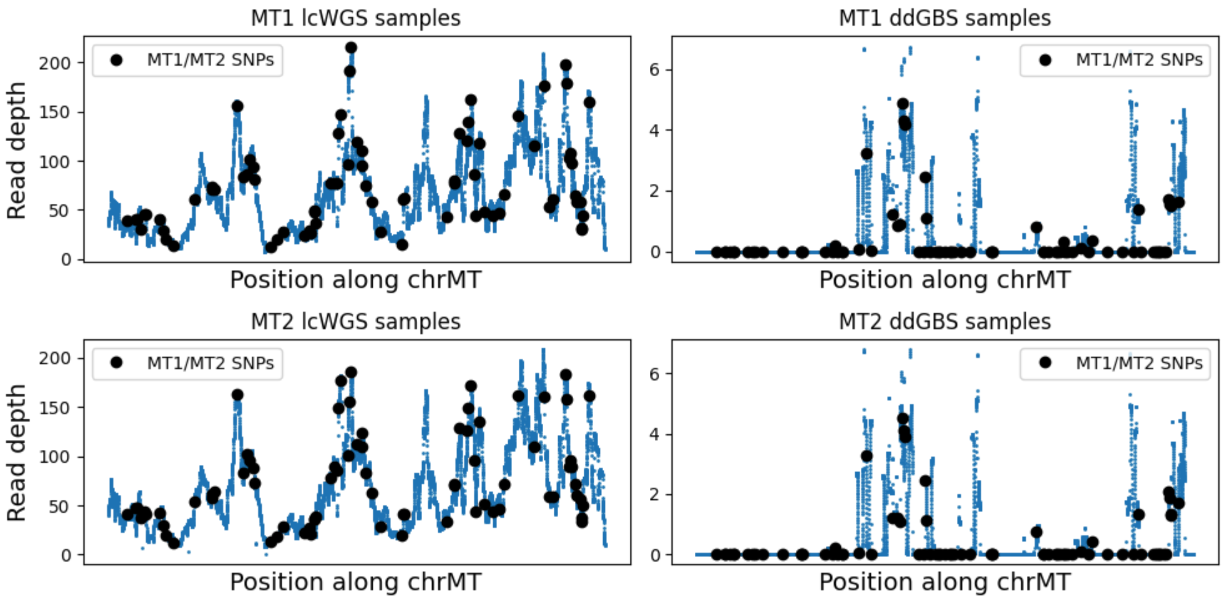

Figure S10. Average read depth in low-coverage data along the MT Chromosome. Plots show position along MT on X-axis and average read depth on Y-axis. Samples split by MT haplotype and by library preparation method. Positions with SNPs between MT1 and MT2 (Figure S6) picked out in large black dots.

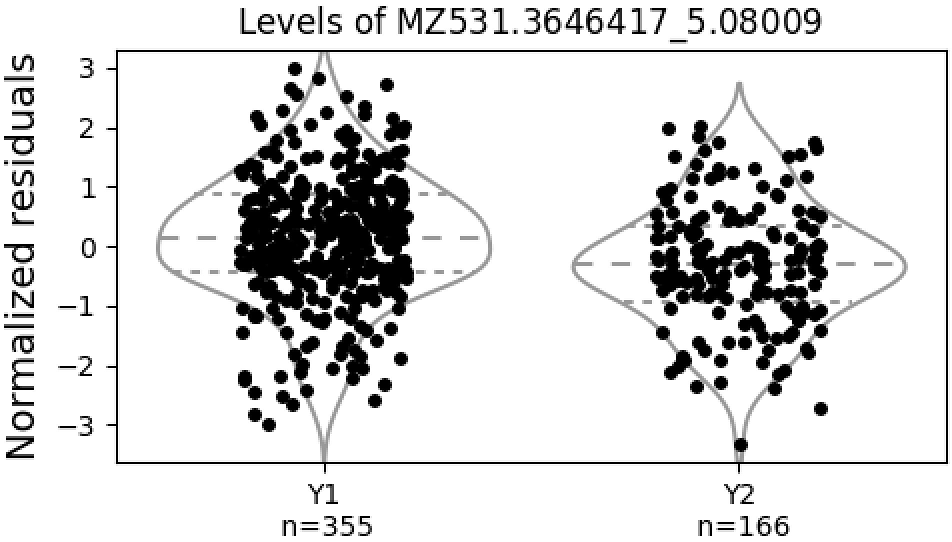

Figure S11. Levels of an unannotated metabolite associated with Y haplogroup. Covariates were regressed out as described in "GWAS phenotype association". Quantile lines included. Plot shows each sample's normalized residuals on Y-axis; samples are split into Y haplogroups on X-axis. Q-value is 0.015.

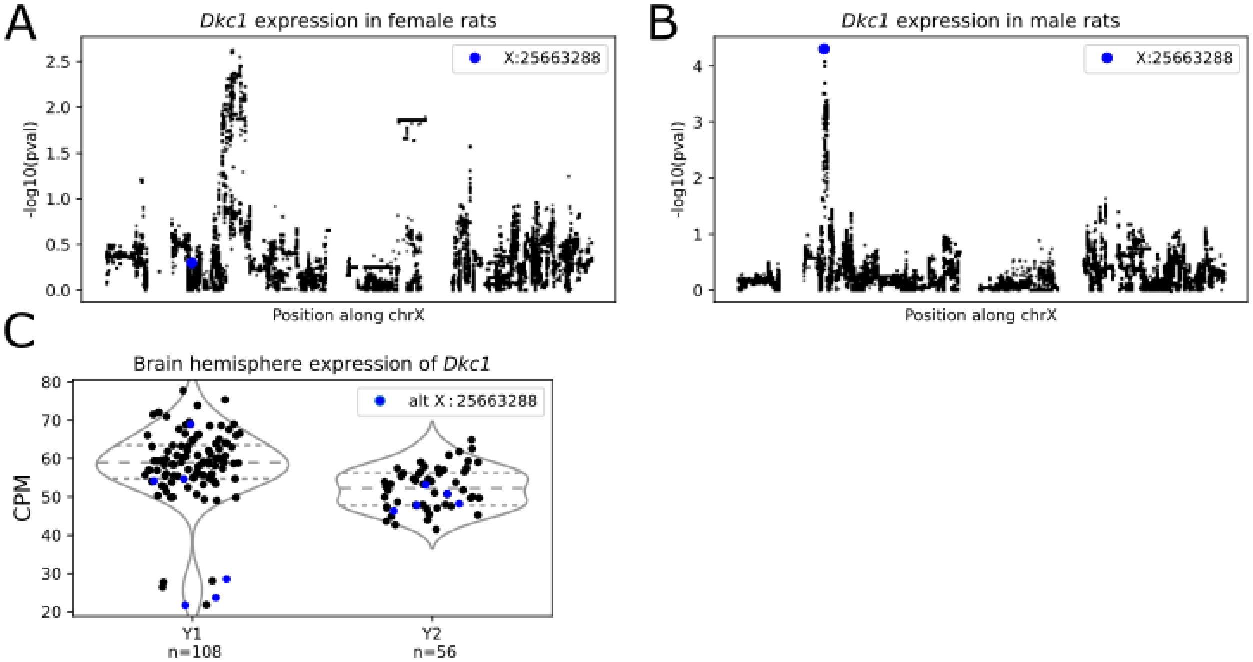

Figure S12. Association between Chromosome X SNPs and *Dkc1* expression. **A-B.** Manhattan plots for association between expression of *Dkc1* in brain hemisphere and genotypes of SNPs across the X Chromosome in **A.** female and **B.** male rats, calculated by a linear model as described in "*Dkc1* expression and X SNPs association". Notably, in male rats, Y haplogroup was used as a covariate. Plot shows position along X on X-axis and p-value -log10 transformed on Y-axis. **C.** Effect plots for the significant *Dkc1* association to show genotype of most-associated SNP. Quantile lines included. Plots show each sample's normalized CPM on Y-axis; samples are split into Y haplogroups on X-axis. Points colored by whether that rat has an alternative allele for the top X SNP. While this SNP is enriched in the Y1 subgroup, both genotypes appear in all groups with no apparent effect. A deletion of nearby *Med14Y* (File S1) is a better explanation for the Y1 subgroup.

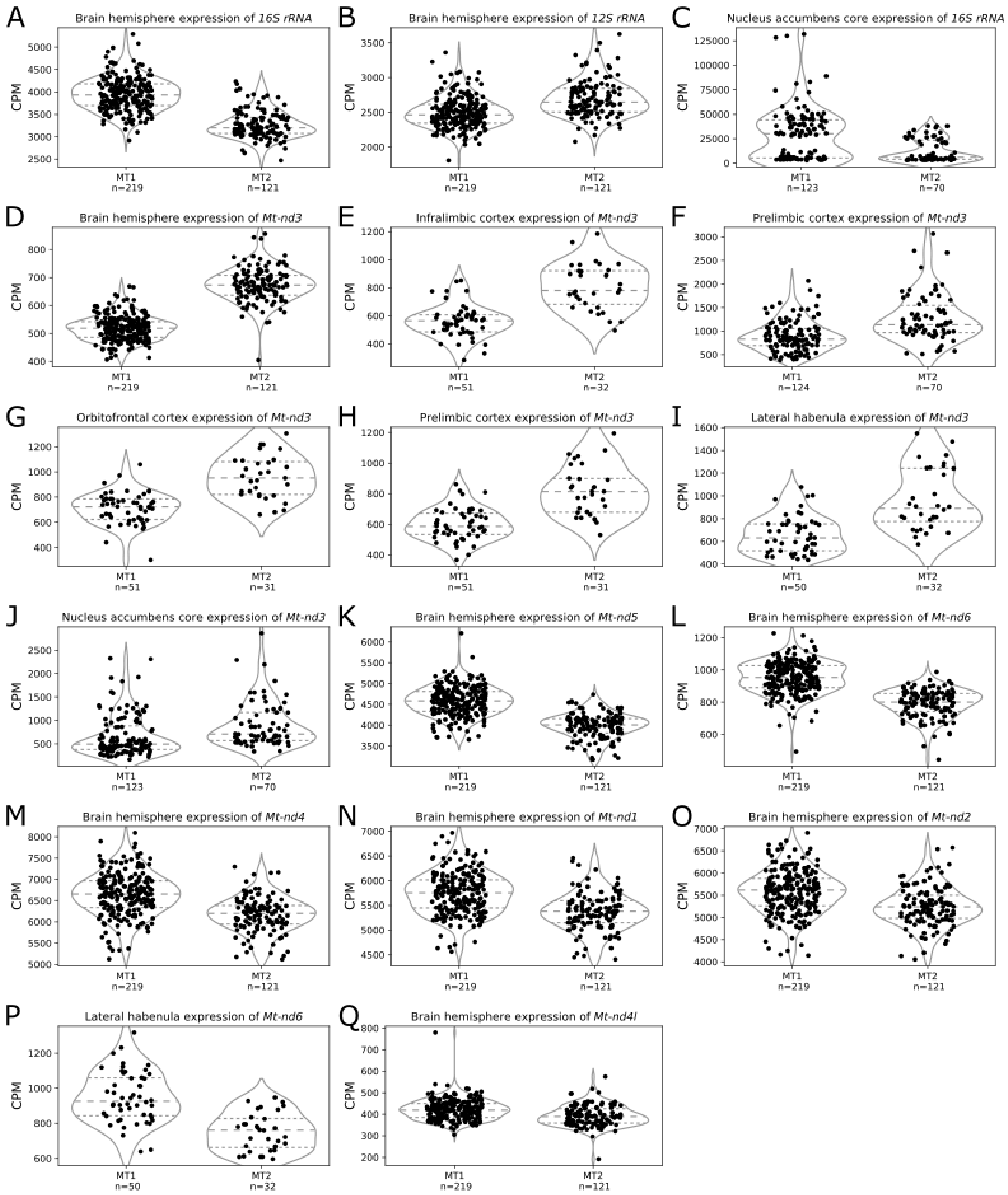

Figure S13. Effect plots for all significant associations between gene expression and MT haplotype. Quantile lines included. Plots show each sample's normalized CPM on Y-axis; samples are split into MT haplotypes on X-axis. P-values of associations are in Table S4. Note that some tissue names are used twice (Table S2). **A-C.** MT-rRNA expression is affected by MT haplotype. **D-J.** *Mt-nd3* is upregulated in MT2 samples **K-Q.** Other Complex I subunits are downregulated in MT2 samples.

project	traits	Y1	Y2	MT1	MT2
Association Between Behavioral Regulation and Cocaine Cue Preference	426	842	462	1598	919
Food and Water Consumption in Heterogeneous Stock Rats	8	286	125	509	308
Genetic Basis of Cecum Metabolome Composition in Heterogeneous Stock Rats	4861	355	166	688	389
Genetic Basis of Cecum Microbiome Composition in Heterogeneous Stock Rats	108	1292	641	2399	1458
Genetic Studies of Incentive Salience	108	532	263	965	610
Genetics of Adiposity Related Traits in Heterogeneous Stock Rats	11*	2246	1128	4115	2510
Genetics Underlying Individual Differences in Skeletal Muscle	4	1183	598	2167	1315
Genomic Analysis of Avoidance Learning in Addiction	9	244	143	671	408
Identification of Genes Regulating Bone Matrix Composition and Quality	60	610	290	1106	632
Identification of Genetic Features of Delay Discounting Using a Heterogeneous Stock Rat Model	29	262	178	537	343
Identification of Genetic Variants that Contribute to Compulsive Cocaine Intake in Rats	48	242	159	464	318
Neurogenetic Substrates of Cocaine Addiction	44	140	90	275	180
Socially-Acquired Nicotine Self-Administration	11	598	299	1088	660
The Genetic Basis of Opioid Dependence Vulnerability in a Rodent Model	28	260	190	525	338
Use of Next-Gen Sequencing to Identify Genetic Variants that Influence Compulsive Oxycodone Intake in Outbred Rats	95	169	100	335	192

Table S1. Summary of PheWAS phenotypes, by original project. Some rats had phenotypes collected for more than one project. Not all rats have a MT haplotype assignment, and not all male rats have a Y haplogroup assignment. Such rats were excluded from analysis. Information about each project is as follows: title, number of traits included in association tests, and number of rats phenotyped by the project who have the Y1, Y2, MT1, and MT2 haplotypes, respectively. Some phenotypes were measured in less than the total number of rats, e.g. due to early death.
* One trait in "Genetics of Adiposity Related Traits in Heterogeneous Stock Rats" was only defined in females, and thus was not included in Y tests.

tissue	n Y1	n Y2	n Y tests	n MT1	n MT2	n MT tests
Basolateral amygdala (BLA)	60	37	19858	123	68	19808
Brain hemisphere (Brain)	108	56	20386	219	121	20437
Eye (Eye)	16	8	18539	35	17	18523
Infralimbic cortex (IL)	29	14	20827	51	32	20822
Lateral habenula (LHb)	28	14	20902	50	32	20957
Nucleus accumbens core (NAcc)	25	13	20704	45	32	20640
Nucleus accumbens core (NAcc2)	59	38	21000	123	70	20866
Orbitofrontal cortex (OFC)	29	14	20650	51	31	20642
Prelimbic cortex (PL)	27	15	20728	51	31	20772
Prelimbic cortex (PL2)	60	37	19905	124	70	19862

Table S2. Summary of gene expression DE tests, by tissue. Datasets from RatGTex (https://ratgtex.org/download/). Shown is number of rats with each haplotype and number of genes where ≥ 10% of haplotyped samples have expression, i.e., the number of samples in each DE group and the number of DE tests conducted. Not all rats have a MT haplotype assignment, and not all male rats have a Y haplogroup assignment. Such rats were excluded from analysis. Some tissues were used in multiple projects. Their data is split by project, e.g. prelimbic cortex is in groups of PL and PL2.

Ensembl ID	gene	tissue	chr	q-value
ENSRNOG00000057231	*Ddx3y*	Brain hemisphere (Brain)	Y	0.000417
ENSRNOG00000055562	*Dkc1*	Brain hemisphere (Brain)	JACYVU010000493.1	0.00125

Table S3. Genes with DE between Y haplogroups (FDR < 0.05). Information about each association is as follows: Ensembl ID (a stable identifier for the Ensembl database) of the gene, common name (from RGD) of the gene, tissue (long name and abbreviation) of the samples, chromosome the gene is on, and BH q-value of the association.

Ensembl ID	gene	tissue	chr	q-value
ENSRNOG00000033615	*Mt-nd3*	Brain hemisphere (Brain)	MT	$1.75 \cdot 10^{-42}$
ENSRNOG00000043866	*16S rRNA*	Brain hemisphere (Brain)	MT	$2.43 \cdot 10^{-32}$
ENSRNOG00000029971	*Mt-nd5*	Brain hemisphere (Brain)	MT	$4.03 \cdot 10^{-30}$
ENSRNOG00000029042	*Mt-nd6*	Brain hemisphere (Brain)	MT	$2.51 \cdot 10^{-29}$
ENSRNOG00000029707	*Mt-nd4*	Brain hemisphere (Brain)	MT	$1.18 \cdot 10^{-12}$
ENSRNOG00000030644	*Mt-nd1*	Brain hemisphere (Brain)	MT	$1.04 \cdot 10^{-9}$
ENSRNOG00000030478	*12S rRNA*	Brain hemisphere (Brain)	MT	$1.52 \cdot 10^{-7}$
ENSRNOG00000031033	*Mt-nd2*	Brain hemisphere (Brain)	MT	$1.05 \cdot 10^{5}$
ENSRNOG00000033615	*Mt-nd3*	Infralimbic cortex (IL)	MT	$5.55 \cdot 10^{-5}$
ENSRNOG00000033615	*Mt-nd3*	Prelimbic cortex (PL2)	MT	$8.59 \cdot 10^{-5}$
ENSRNOG00000033615	*Mt-nd3*	Orbitofrontal cortex (OFC)	MT	0.000210
ENSRNOG00000033615	*Mt-nd3*	Prelimbic cortex (PL)	MT	0.000318
ENSRNOG00000029042	*Mt-nd6*	Lateral habenula (LHb)	MT	0.000477
ENSRNOG00000031053	*Mt-nd4l*	Brain hemisphere (Brain)	MT	0.000477
ENSRNOG00000033615	*Mt-nd3*	Lateral habenula (LHb)	MT	0.00438
ENSRNOG00000043866	*16S rRNA*	Nucleus accumbens core (NAcc2)	MT	0.00679
ENSRNOG00000033615	*Mt-nd3*	Nucleus accumbens core (NAcc2)	MT	0.0146

Table S4. Genes with DE between MT haplotypes (FDR < 0.05). Information about each association is as follows: Ensembl ID (a stable identifier for the Ensembl database) of the gene, common name (from RGD) of the gene, tissue (long name and abbreviation) of the samples, chromosome the gene is on, and BH q-value of the association.

	1 kidney	2 kidneys
MT1 (BN)	73	3365
MT2 (nearly ACI)	35	2058

Table S5. Contingency table of number of kidneys at birth and MT haplotype, for all rats where both are known. The one-sided p-value for a Fisher's exact test is 0.14 for association between MT haplotype and number of kidneys.
